# Clinical and Epidemiological Characteristics of COVID-19 Patients in Chongqing China

**DOI:** 10.3389/fpubh.2020.00244

**Published:** 2020-05-29

**Authors:** Ao Yang, Qian Qiu, Xianghua Kong, Yanyu Sun, Tingying Chen, Yujie Zuo, Danfeng Yuan, Wei Dai, Jihong Zhou, Anzhou Peng

**Affiliations:** ^1^Department of Traffic Injury Prevention Research Office, Army Medical Center of the PLA, Daping Hospital, Chongqing, China; ^2^Department of Tuberculosis Research Office, Chongqing Public Health Medical Treatment Center, Chongqing, China; ^3^Department of the Fifth Tuberculosis, Chongqing Public Health Medical Treatment Center, Chongqing, China; ^4^Department of the Third Infection, Chongqing Public Health Medical Treatment Center, Chongqing, China; ^5^Department of General Internal Medicine, Chongqing Public Health Medical Treatment Center, Chongqing, China; ^6^Department of the Second Tuberculosis, Chongqing Public Health Medical Treatment Center, Chongqing, China

**Keywords:** SARS-Cov-2, COVID-19, epidemiological, clinical characteristics, China

## Abstract

**Objectives:** To study in-depth the clinical and epidemiological characteristics of pneumonia resulting from COVID-19 and provide evidence for effective public health decisions.

**Methods:** This was a retrospective, single-center research study. Participants were enrolled from patients presenting at the Chongqing Public Health Medical Treatment Center from Jan 24 to Feb 7, 2020, and were confirmed as having COVID-19.

**Results:** A total of 114 COVID-19 patients (99 mild, 4 severe, 11 critical) of which 56 (56/114; 49.1%) were male, 58 (58/114; 50.9%) were female with a mean age of 46.05 years. Twenty nine (29/114; 25.44%) patients suffered from chronic diseases. Neutrophils counts in 23.68% (27/114) of patients were abnormally low and abnormally high in 21.05% (24/114). Erythrocyte sedimentation rate and the C-reactive protein levels were abnormally elevated in 76.5% (62/81) and 62.9% (66/105) of patients, respectively. Creatine kinase isoenzymes (CK-MB), pro-brain natriuretic peptide (pro-BNP) and troponin levels were above the normal range in 7.10% (8/112), 66.7% (10/15), and 100% of patients, respectively. The percentage of patients in which the partial pressure of oxygen (PaO_2_)/fraction of inspired O_2_(FiO_2_) ratio exceeded 200 was 60%. A total of 91 (91/114; 79.82%) patients displayed severe bilateral pneumonia, 52 (52/114; 45.61%) exhibited ground-glass opacity, and pulmonary consolidation was observed in 4 (3.51%) patients. Differences in shortness of breath, insomnia, inappetence, the procalcitonin (PCT) levels, FiO_2_ and PaO_2_/FiO_2_ among the three groups were statistically significant (*p* < 0.05). Differences between the mild and severe groups was observed in neutrophil and lymphocyte counts, CD4 expression, and levels of C-reactive protein, alanine aminotransferase, aspartate aminotransferase and albumin (*P* < 0.05). Between the mild and critical groups, differences were observed in neutrophils, platelets, and CD4 expression (*P* < 0.05). A difference in C-reactive protein levels between severe and critical groups was also found (*P* < 0.05).

**Conclusions:** In the majority of cases no gender differences were observed and mostly the symptoms were mild. Evidence of efficient human-to-human virus transmission was found. The elderly with comorbidities were more prone to develop into severe or critical illness. Age and comorbidity may be risk factors for poor outcome.

## Introduction

As of December of 2019, a new coronavirus disease (COVID-19) caused by the SARS-CoV2 virus emerged in Wuhan, Hubei Province, China, and has spread globally via travel (1, 2). The pandemic was initially declared a Public Health Emergency of International Concern on January 20th, 2020 by the World Health Organization (WHO) (1, 3). By February 17th, there had been 72,436 confirmed cases and 1,868 confirmed deaths in Chinese Provinces and affiliated entities as of, according to the Chinese Healthy Authority, and 332,930 confirmed cases globally as of March 23rd, 2020, there being no antiviral treatments so far proven to be efficacious (4). Efforts to contain the pandemic have instead focused on public health measures such as social distancing, prohibition of public gatherings, and increased use of face masks. These measures alone, however, are unlikely to stop the pandemic owing to the highly contagious nature of the disease (5).

Chongqing Public Health Medical Center, a designated treatment hospital for such patients, received nearly 200 COVID-19 patients. To facilitate diagnosis and treatment, additional clinical and epidemiological features of SARS-CoV-2 pneumonia are required (6–8). Therefore, a study of the clinical and epidemiology features of 114 patients admitted to Chongqing Public Health Medical Center confirmed to have COVID-19, was conducted.

## Methods

### Study Design

A retrospective, single-center study was conducted. All participants presented at the Chongqing Public Health Medical Treatment Center (the designated treatment hospital for COVID-19 patients), from Jan 24 to Feb 7, 2020.

All presenting subjects diagnosed with COVID-19 in accordance with the “COVID-19 Prevention and Control Plan (Sixth Edition)” were enrolled in the study. They were confirmed using two methods, real-time RT-PCR (from an upper respiratory tract throat swab) and the chest computed tomography (CT) scan. The Chinese Center for Disease Control (CDC) was the source of RT-PCR detection reagents.

In accordance with the “COVID-19 Prevention and Control Plan (Sixth Edition),” patients were categorized by the severity of COVID-19 into three groups: mild, severe, or critical. Biochemical indicators were compared between groups, then analyzed. Epidemiological, demographic, clinical, and laboratory information were obtained from patients' medical records. Three physicians reviewed all data.

The epidemiological data of all patients (i.e., history of exposure to wildlife, history of travel or work in the epidemic area of Wuhan), confirmed patient contact history and details of family grouping; demographics (i.e., sex, age, work, period since onset, period since diagnosis and treatment), symptoms upon admission, basic disease, laboratory results, chest radiographic/CT findings and treatments that had been administered for severe COVID-19, were recorded. A comparison of these data was conducted among the mild, severe and critical patient groups.

The study information has filtered the patient's personal identity and other private information and therefore the requirements for ethical approval and informed consent were waived by the relevant authorities for the purposes this study. Access to the data was provided to the authors under permission by the Medical Administration Division of Chongqing Public Health Medical Treatment Center.

### Statistical Analysis

Continuous measurements that were normally distributed are expressed as means (SD). Median (inter quartile range [IQR]) values were used to express continuous variables that were not normally distributed. Categorical variables were presented as count (%). Laboratory results outside the normal range were included. SPSS software was used for statistical testing. Count data were compared using a Chi-squared test, while measured data were compared using an ANOVA or Student's *t*-test as appropriate. Variance was evaluated using a Kruskal-Wallis test. All statistical testing was two-sided, with *P* < 0.05 considered statistically significant. Study data are expressed as means ± SD.

## Results

### Overall Analysis Results of 114 Patients

A total of 114 COVID-19 patients (99 mild, 4 severe, 11 critical; Table 1) were included in the study. Of these, 56 (56/114; 49.12%) were male and 58 (58/114; 50.87%) female. The mean age was 46.05 years (SD: 15.15; Table 1). A total of 44 (44/114; 38.60%) patients had a history of travel to Wuhan, 1 (1/114; 0.90%) patient was exposed to wildlife, 66 (66/114; 57.89%) patients had a history of close contact with COVID-19 patients, and 48 (48/114; 42.11%) patients lived in a close family grouping.

**Table 1:** Demographics, baseline characteristics and severity grade of COVID-19 of 114 patients admitted to Chongqing Public Health Medical Treatment Center (Jan-18, 2020) with COVID-19.

**Demographics, baseline characteristics**		**Patients (*n =* 114)**
**Severity grade**		
	Mild	99 (86.84%)
	Severe	4 (3.51%)
	Critical	11 (9.65%)
**Sex**	Male	56 (49.12%)
	Female	58 (50.88%)
**Age, years**		
Mean (SD)	46.5 (15.15)	
Range	10–77	
	7–13	2 (1.75%)
	13–18	2 (1.75%)
	18–25	7 (6.14%)
	25–65	91 (79.82%)
	>65	12 (10.53%)
**Occupation**	Civil servant/teacher/retired	12 (10.53%)
	Worker	6 (5.26%)
	Farmer	12 (10.53%)
	Self-employed	7 (6.14%)
	Student	6 (5.26%)
	Company employee	17 (14.91%)
	No job	19 (16.67%)
	Unknown	35 (30.70%)
**Chronic medical illness**		
	Cardiovascular and cerebrovascular diseases	12 (10.53%)
	Urinary system disease	1 (0.88%)
	Endocrine system disease	12 (10.53%)
	Respiratory system disease	1 (0.87%)
	Digestive system disease	3 (2.63%)

The occupations of patients were diverse. Company employees accounted for 14.91% (17/114). Twenty nine (29/114; 25.44%) patients had chronic diseases, mostly cardiovascular, endocrine, or cerebrovascular conditions (Table 1).

The symptoms at admission included mainly fever, cough, sputum, inappetence and dyspnea. Other symptoms are displayed in Table 2. All patients enrolled in the study accepted symptomatic treatment. Mechanical ventilation (8 non-invasive, 3 invasive) was used to treat the 11 critical patients.

**Table 2:** Clinical characteristics of critical COVID-19 patients.

**Clinical characteristics**	**Patients *n =* 114**
**The time interval from onset to the first diagnosis**	2.34 (2.13)
**The time interval from the first diagnosis to hospitalization**	2.18 (1.75)
**Symptoms at admission**	
Fever	13 (19.40%)
Cough	72 (63.16%)
Sputum	33 (28.95%)
Dyspnea	17 (14.91%)
Nasal congestion	7 (0.61%)
Rhinorrhoea	6 (5.26%)
Diarrhea	7 (0.61%)
Nausea and vomiting	5 (4.39%)
Insomnia	7 (0.61%)
Inappetence	22 (19.30%)
Frequent urination	2 (1.75%)
Headache	1 (0.88%)
Sore throat	1 (0.88%)

The mean (SD) time interval from onset to first diagnosis was 2.34 (2.13) days, while from first diagnosis to hospitalization it was 2.18 (1.75).

Body temperature was measured in 67 patients and ranged from 37.2 to 39.2°C. The temperature exceeded 38.5°C in 13 (13/67; 19.40%).

On admission, the number of neutrophils was abnormally low in 23.70% (27/114) of patients, and abnormally elevated in 21.90% (24/114) (Table 3). In many patients, numbers of leucocytes, lymphocytes, platelets, hemoglobin content, and CD4 expression were below the normal range (Table 3).

**Table 3:** Laboratory results of critical COVID-19 patients.

**Laboratory index**		**Patients (*n =* 114)**
**Blood routine (Units; normal range)**		
Leucocytes (× 10^9^ per L; 3.5–9.5)		5.15 (2.11)
	Above	4.40%
	Below	15.80%
Neutrophils granulocyte (%; 50–70)		60.20 (12.78)
	Above	21.05%
	Below	23.68%
Lymphocytes (× 109 per L; 1.1–3.2)		1.52 (0.99)
	Above	0.88%
	Below	24.56%
Platelets (× 109 per L; 125.0–350.0)		134.38 (15.97)
	Above	6.14%
	Below	21.05%
Hemoglobin (g/L; 130.0–175.0)		188.25 (88.03)
	Below	39.47%
Cluster of differentiation 4 (per μl; 500–1,600)		436.56 (236.53)
	Below	62.50%
**Blood biochemistry**		
Albumin (g/L; 40.0–55.0)		41.52 (4.23)
	Below	30.70%
ALT (U/L; 9.0–50.0)		29.99 (29.39)
	Above	13.16%
	Below	3.51%
AST (U/L; 15.0–40.0)		29.39 (21.46)
	Above	12.28%
	Below	3.51%
Total bilirubin (μmol/L; 0.0–21.0)		15.71 (10.09)
	Above	20.18%
BUN (mmol/L; 3.6–9.5)		3.89 (1.25)
	Below	45.61%
Serum creatinine (μmol/L; 57.0–111.0)		69.76 (20.09)
	Below	27.19%
Creatine kinase (U/L; 50.0–310.0)		111.46 (169.36)
	Above	5.36%
	Below	27.68%
**Infection-related biomarkers**		
Procalcitonin (ng/mL; 0.0–5.0)		0.06 (0.12)
Erythrocyte sedimentation rate (mm/h; 0.0–15.0)		38.37 (25.36)
	Above	76.54%
CRP (mg/L; 0.0–5.0)		20.08 (29.38)
	Above	62.86%
**Myocardial injury markers**		
Creatine kinase (U/L; 50.0–310.0)		111.46 (169.36)
	Above	5.36%
	Below	27.68%
Creatine kinase isoenzymes, CK-MB (U/L; 0–18)		11.25 (9.85)
	Above	7.14%
Pro-brain natriuretic peptide (pg/mL; normal range <100)		837.51 (1580.18)
	Above	66.67%
Troponin (μg/L; <0.2)		9.88 (5.66)
	Above	100.00%
**Blood gas analysis**		
pH (7.35–7.45)		7.44 (0.07)
	Above	32.00%
	Below	68.00%
PO_2_ (10.64–13.3 kPa or 80–100 mmHg)		81.32 (23.14)
	Above	20.00%
	Below	56.00%
PCO_2_ (4.65–5.98 kPa or 35–45 mmHg)		42.32 (13.02)
	Above	28.00%
	Below	26.00%
Fraction of inspiration O_2_, FiO_2_		36.92 (15.97)
PO_2_/FiO_2_		262.56 (109.93)
	>200	60.00%

Albumin, blood urea nitrogen and serum creatinine were below the normal range in many patients, but total bilirubin was above the normal range. Alanine aminotransferase (ALT) and aspartate aminotransferase (AST) were mostly above the normal range, but the creatine kinase was generally below the normal range in many patients.

Procalcitonin (PCT) levels were normal in all cases, but erythrocyte sedimentation rate and C-reactive protein (CRP) levels were above normal in 76.5% (62/81) and 62.9% (66/105) of patients, respectively. Myocardial injury markers such as creatine kinase isoenzymes (CK-MB), pro-brain natriuretic peptide (pro-BNP) and troponin were above the normal range in 7.10% (8/112), 66.7% (10/15), and 100% of patients respectively.

PaO_2_ as measured by blood gas analysis was low in 56% (14/25) of patients. Blood pH was above the normal range in 44% (8/25) of patients. PaCO_2_ was both above and below the normal range in 20% (5/25) and 56% (14/25) of patients, respectively. The percentage of patients in whom the PaO_2_/FiO_2_ ratio exceeded 200 was 60% (15/25) (Table 3).

From CT chest scans, 91 (91/114; 79.82%) patients exhibited severe bilateral pneumonia, 52 (52/114; 45.61%) patients displayed ground-glass opacity, and 4 (4/114; 3.51%) patients showed pulmonary consolidation.

### Comparison of Patient Characteristics in the Different Patient Groups by Severity of COVID-19 Symptoms

Differences in sex, age group, interval from onset to first diagnosis and first diagnosis to hospitalization, and exposure history was not statistically significant between the mild, severe, and critical group patients (*p* > 0.05). The mean (SD) age of the three groups was 44 years (14.62), 63.75 years (9.67), and 58.09 years (12.03), respectively. The differences between the mild group and the other two groups were statistically significant (*p* < 0.001). The greatest difference in number in each occupation between the three groups was between the mild and the critical groups (*p* < 0.001). Of all signs and symptoms at admission, only differences in shortness of breath, insomnia, and inappetence were statistically significant, and that was true for all three groups (*p* < 0.001; Table 4).

**Table 4:** Comparison results of demographics, baseline characteristics of different severity grade COVID-19 patients.

**Demographics, baseline characteristics**		**Mild (*n =* 99)**	**Severe (*n =* 4)**	**Critical (*n =* 11)**	***P***
Sex	Male	49	0	7	0.09
	Female	50	4	4	
Age	Mean (*SD*)	44 (14.62)^a^^b^	63.75 (9.67)	58.09 (12.03)	<0.05
	7~13	2	0	0	0.14
	13~18	2	0	0	
	18~25	7	0	0	
	25~65	81	2	8	
	>65	7	2	3	
Interval of onset-first diagnosis	Mean (*SD*)	2.31 (2.15)	1.75 (1.71)	2.82 (2.18)	0.59
Interval of first diagnose-hospitalization	Mean (*SD*)	2.12 (1.73)	2.75 (2.87)	2.45 (1.64)	0.67
Occupation	Civil servant/teacher/retired	7^b^	1	4	0.05
	Worker	5^b^	0	1	
	Farmer	11^b^	1	0	
	Self-employed	7^b^	0	0	
	Student	6^b^	0	0	
	Company employee	16^b^	0	1	
	No job	13^b^	2	4	
	Unknown	34^b^	0	1	
Exposure history	Wildlife exposure	1	0	0	0.93
	Wuhan travel history	40	1	3	0.59
	COVID-19 patient close contact history	59	3	4	0.26
	Aggregation	43	2	3	0.56
Signs and symptoms at admission	Fever	13	0	0	
	Cough	64	3	5	0.41
	Sputum	28	2	3	0.64
	Shortness of breath	8^a^^b^	3^c^	6	<0.001
	Nasal congestion	5	0	2	0.22
	Rhinorrhoea	5	0	1	0.76
	Diarrhoea	6	0	1	0.81
	Nausea and vomiting	4	0	1	
	Insomnia	3^a^^b^	0^c^	4	<0.05
	Inappetence	17^a^^b^	0^c^	5	0.048
	Frequent urination	9	1	1	0.57

a Difference between mild group and severe groups had statistical significance (p < 0.05).

b Difference between the mild and the critical groups had statistical significance (p < 0.05).

c *Difference between the severe and the critical groups had statistical significance (p < 0.05)*.

Levels of procalcitonin (PCT), fraction of inspired O_2_ (FiO_2_), and the PaO_2_/FiO_2_ ratio among the different patient groups were significantly different (*P* < 0.05). In addition, the differences in numbers of neutrophils, lymphocytes, CD4 expression, and levels of C-reactive protein, alanine aminotransferase, aspartate aminotransferase and albumin observed in the mild group compared with the severe group were significant (*P* < 0.05). Compared with the mild group, the difference in numbers of neutrophils, platelets, and CD4 expression in the critical group was significant (*P* < 0.05). A difference in C-reactive protein levels was evident between the severe and critical groups (*P* < 0.05), as presented in Table 5.

**Table 5:** Comparison results of laboratory of different severity grade patients with COVID-19.

**Laboratory index**		**Mild (*n =* 99)**	**Severe (*n =* 4)**	**Critical (*n =* 11)**	***P***
Blood routine	Leucocytes (× 10^9^ per L; 3.5–9.5)	5.01 (1.83)	6.62 (2.79)	5.90 (3.66)	<0.001
	Neutrophils granulocyte (%; 50–70)	58.06 (11.56)^a^^b^	71.73 (7.02)	75.28 (13.19)	<0.001
	Lymphocytes (× 10^9^ per L; 1.1–3.2)	1.60 (1.02)^a^	1.25 (0.35)	0.87 (0.44)	>0.05
	Platelets (× 10^9^ per L; 125.0–350.0)	185.38 (80.21)^b^	313.50 (146.28)	168.45 (106.32)	0.047
	Hemoglobin (g/L; 130.0–175.0)	134.77 (16.417)	120.75 (9.98)	135.82 (11.55)	>0.05
	Cluster of differentiation 4 (per μl; 500–1,600)	475.49 (235.02)^a^^b^	251.00 (185.06)	237.40 (160.80)	<0.001
Blood biochemistry	Albumin (g/L; 40.0–55.0)	42.07 (4.01)^a^	38.08 (6.12)	37.91 (3.30)	0.003
	Alanine aminotransferase (U/L; 9.0–50.0)	28.06 (28.02)^a^	27.25 (19.26)	48.36 (39.30)	0.045
	Aspartate aminotransferase (U/L; 15.0–40.0)	27.70 (19.14)^a^	28.50 (15.07)	44.91 (35.41)	0.003
	Total bilirubin (μmol/L; 0.0–21.0)	15.39 (10.27)	15.05 (8.50)	18.86 (9.17)	>0.05
	Blood urea nitrogen (mmol/L; 3.6–9.5)	3.84 (1.23)	4.68 (1.63)	4.08 (1.30)	>0.05
	Serum creatinine (μmol/L; 57.0–111.0)	69.84 (20.61)	69.63 (9.16)	69.15 (19.28)	>0.05
	Creatine kinase (U/L; 50.0–310.0)	88.80 (64.93)	201.00 (202.43)	278.73 (476.09)	>0.05
Infection-related biomarkers	Procalcitonin (ng/mL; 0.0–5.0)	0.05 (0.06)^a^^b^	0.02 (0.00)^c^	0.16 (0.32)	0.014
	Erythrocyte sedimentation rate (mm/h; 0.0–15.0)	36.72 (26.11)	59.33 (24.54)	43.30 (17.75)	>0.05
	C-reactive protein (mg/L; 0.0–5.0)	16.75 (26.98)^a^	7.56 (3.62)^c^	51.86 (34.94)	<0.001
Myocardial injury markers	Creatine kinase (U/L; normal range 50.0–310.0)	88.80 (64.93)	201.00 (202.43)	278.73 (476.09)	>0.05
	Creatine kinase isoenzymes, CK-MB (U/L; normal range 0–18)	11.12 (10.18)	10.20 (3.13)	12.75 (8.69)	>0.05
	pro-Brain Natriuretic Peptide (pg/mL; normal range <100)	1705.28 (2326.45)	170.40 (0.00)	270.06 (225.67)	>0.05
	Troponin (μg/L; normal range <0.2)	14.05 (6.58)	8.25 (2.71)	6.35 (2.10)	>0.05
Blood gas analysis	PH (normal range 7.35–7.45)	7.42 (0.07)	7.44 (0.07)	7.47 (0.06)	>0.05
	PO_2_ (normal range 10.64–13.3 kPa or 80–100 mmHg)	90.90 (23.81)	83.75 (25.41)	71.73 (19.66)	>0.05
	PCO_2_ (normal range 4.65-5.98 kPa or 35–45 mmHg)	43.70 (4.81)	41.50 (7.05)	41.36 (19.19)	>0.05
	Fraction of inspiration O_2_, FiO_2_	24.00 (4.92)^a^^b^	33.50 (9.29)^c^	49.91 (14.38)	<0.001
	PO_2_/FiO_2_	376.90 (46.89)^a^^b^	252.00 (53.39)^c^	162.45 (45.03)	<0.001

a Difference between the mild and severe groups had statistical significance (p < 0.05).

b Difference between the mild and critical groups had statistical significance (p < 0.05).

c *Difference between the severe and critical groups had statistical significance (p < 0.05)*.

## Discussion

The present study reported on almost all hospitalized COVID-19 patients to date in Chongqing, and recorded the epidemiological and clinical features of the disease. Of all 114 hospitalized COVID-19 patients, 99 were classified as mild, 4 as severe, and 11 as critical. The mean time interval from onset to first diagnosis was 2.34 and 2.18 days from first diagnosis to hospitalization. Common symptoms included fever, cough, sputum, inappetence and shortness of breath. However, a significant proportion of patients initially presented with atypical symptoms, such as nausea, vomiting, and frequent urination. According to chest CT scans, 91 (79.82%) patients displayed bilateral pneumonia. Multiple mottling and ground-glass opacity is a hallmark of chest CT scans for COVID-19. Laboratory features included abnormalities of hemocytes, depressed CD4, expression, hemoglobin and PaO_2_, in addition to elevated total bilirubin, ALT, AST, pro-BNP, and troponin. The majority of critical patients were older and had a greater number of underlying medical conditions than did mild patients.

No statistically significant gender bias was experienced in the patients enrolled in this study, consistent with a report evaluating 138 patients hospitalized in Zhongnan Hospital in Wuhan between January 1 and 28 (9). However, these data differ from early reports that indicated that SARS-CoV-2 infection was more likely to affect males (8, 10). A possible explanation is that cases in previous studies were from the earliest phase of local outbreak and the subsequent phase of the local epidemic, mostly related to exposure associated with the Huanan Seafood Wholesale Market, most workers of which were males. The cases described herein were from the widespread national outbreak stage (9). In the current stage, no male dominance among the patients was evident, with fewer patients having been exposed to the Seafood Market (11). Furthermore, the mean age of COVID-19 patients presenting in the present study was 46.5 years. This result is consistent with the research of Yang Yao in Shaanxi province outside Wuhan (12). In that study, the majority were mild cases (86.84%), and only 9.6% required a ventilator, far fewer than those reported in prior studies from the early stages of the COVID-19 pandemic (11), indicating that this disease has potentially become more mild as the chains of transmission have grown. However, the majority of COVID-19 cases, including those which are mild, can still quickly become severe or critical without medical support (13).

A number of studies have suggested that rapid person-to-person transmission of SARS-CoV-2 was the major mode of transmission (9, 14). The data in this study indicate a history of high family grouping (42.1%) and close contact with COVID-19 patients (57.9%). These data are evidence of efficient human-to-human viral transmission. A reason for the rapid spread may due to abundant routes of transmission. According to the sixth edition of guidance for diagnosis and treatment of COVID-19 issued by the National Health Commission of China, SARS-CoV-2 is transmitted through respiratory aspirates, droplets, contact and feces, and even vertical transmission (15). Another explanation may be atypical symptoms in the early stages of SARS-CoV-2 infection. From data-driven analysis, the basic reproduction number in the early stage ranged from 2 to 3.5, such that each case was linked to 2–3.5 new infections (16–19). Government-implemented quarantine efforts are thus required for the control of further COVID-19 outbreaks.

A number of changes in symptomatology have already been observed in patients in Chongqing. The presenting symptoms included fever (19.4%), cough (63.16%), sputum (28.95%), inappetence (19.30%), and shortness of breath (14.91%), followed by nasal congestion, rhinorrhea, diarrhea, nausea, and vomiting, insomnia, frequent urination, headache and sore throat. In comparison, fever was observed in more than 90% of hospitalized patients (9, 10, 14). We speculate that increases in confirmed asymptomatic cases were due to the wide use of RT-PCR fast detection technology and intense surveillance. Compared with symptoms in mild patients, more common in severe or critical patients included shortness of breath, insomnia, and inappetence. Symptom onset may allow physicians to identify patients with poor prognosis.

Individuals suffering from viral pneumonia typically exhibit low oxygen saturation, deviations in blood gas levels, and clear abnormalities in chest imaging scan including areas of patchy consolidation, ground glass opacity, alveolar exudate, and interlobular involvement (20). The present study found similar chest imaging results. The most common laboratory abnormalities observed in the present study were depressed numbers of leucocytes, lymphocytes, lower hemoglobin content, platelets, CD4 expression and PaO_2_, and reduced levels of albumin, blood urea nitrogen, serum creatinine, and creatine kinase, in addition to elevated total bilirubin, ALT, AST, pro-BNP, and troponin. These laboratory abnormalities are consistent with prior findings in those with SARS-CoV2 infection (14), and even MERS-CoV and SARS-CoV infection (9), suggesting that SARS-CoV-2 infection may be associated with cellular immunodeficiency, myocardial and hepatic injury, nutrient consumption and hypoxemia. Compared with patients with mild symptoms, those with severe or critical symptoms exhibited differences in neutrophil count and CD4 expression. Differences in CRP levels were also found in severe and critical groups (Table 5). The difference in neutrophil count and CRP may be related to the cytokine storm induced by virus invasion of the virus. These parameters possibly predict disease severity and represent potential biomarkers.

The present study also indicated that patients in severe and critical groups were mostly elderly, the majority with comorbidities prior to admission. This suggests that age and comorbidities are linked to risk of poor outcome. Wang et al. found that the median time from onset of symptoms to death in persons aged 70+ was 11.5 days, significantly lower than in younger individuals (20 days) (21). These findings suggest the disease may progress faster in the elderly than in the young. In addition to older individuals being more likely to be infected with SARS-CoV-2 (16). From the above, although a definitive association could not be made, attention should be paid to the elderly as they may be more vulnerable to the SARS-CoV-2 (21).

The duration of the study was relatively short (January 24th to February 7th, 2020), studying a sudden outbreak of an infectious disease, of which only confirmed cases during this period were examined. Thus, the small sample size, and lack of additional centers within the study were limitations. Despite these limitations, some specific conclusions can be drawn, from which subsequent studies should be built.

In this single-center case series of 114 hospitalized patients with confirmed COVID-19 in Chongqing, China, epidemiological and clinical features were recorded, which may help provide guidance for frontline medical staff in the clinical management of the outbreak. However, the present study is limited by its geographical location and the insufficient sample size. A large-scale multicenter study is required to verify our findings.

## Data Availability Statement

The datasets used and/or analyzed during the current study are available from the corresponding author on reasonable request.

## Ethics Statement

The study information has filtered the patient's personal identity and other private information and therefore the requirements for ethical approval and informed consent were waived by the relevant authorities for the purposes this study. Access to the data was provided to the authors under permission by the Medical Administration Division of Chongqing Public Health Medical Treatment Center.

## Author Contributions

AY, QQ, and AP were involved in the conception and design of the study. AY performed the data analysis, had full access to all of the study data, and take responsibility for the integrity of the data and the accuracy of the analyses. XK, YS, TC, and YZ were responsible for collecting and organizing all patients' data. DY and WD were responsible for aggregating data. JZ was in charge of guiding our research design. AY and QQ drafted the manuscript. All authors were involved in the interpretation of the results, made major contributions to critically reviewing the manuscript for important intellectual content, and read and approved the final manuscript.

## Conflict of Interest

The authors declare that the research was conducted in the absence of any commercial or financial relationships that could be construed as a potential conflict of interest.

## Abbreviations

COVID-19: Corona Virus Disease 2019
SD: standard deviation
RT-PCR: Reverse Transcription-Polymerase Chain Reaction
CRP: C-reactive protein
CK-MB: creatine kinase isoenzymes
pro-BNP: pro-brain natriuretic peptide
PCT: procalcitonin
CD4: cluster of differentiation 4
WHO: World Health Organization
CDC: Center for Disease Control
ALT: alanine aminotransferase
AST: aspartate aminotransferase
CT: Computed Tomography
PH: hydrogen ion concentration
FiO_2_: fraction of inspiration O_2_.
